# Mycolactone Diffuses from *Mycobacterium ulcerans*–Infected Tissues and Targets Mononuclear Cells in Peripheral Blood and Lymphoid Organs

**DOI:** 10.1371/journal.pntd.0000325

**Published:** 2008-10-22

**Authors:** Hui Hong, Emmanuelle Coutanceau, Marion Leclerc, Laxmee Caleechurn, Peter F. Leadlay, Caroline Demangel

**Affiliations:** 1 University of Cambridge, Department of Biochemistry, Cambridge, United Kingdom; 2 Institut Pasteur, UP Pathogénomique Mycobactérienne Intégrée, Paris, France; University of Tennessee, United States of America

## Abstract

**Background:**

Buruli ulcer (BU) is a progressive disease of subcutaneous tissues caused by *Mycobacterium ulcerans*. The pathology of BU lesions is associated with the local production of a diffusible substance, mycolactone, with cytocidal and immunosuppressive properties. The defective inflammatory responses in BU lesions reflect these biological properties of the toxin. However, whether mycolactone diffuses from infected tissues and suppresses IFN-γ responses in BU patients remains unclear.

**Methodology/Principal Findings:**

Here we have investigated the pharmacodistribution of mycolactone following injection in animal models by tracing a radiolabeled form of the toxin, and by directly quantifying mycolactone in lipid extracts from internal organs and cell subpopulations. We show that subcutaneously delivered mycolactone diffused into mouse peripheral blood and accumulated in internal organs with a particular tropism for the spleen. When mice were infected subcutaneously with *M. ulcerans*, this led to a comparable pattern of distribution of mycolactone. No evidence that mycolactone circulated in blood serum during infection could be demonstrated. However, structurally intact toxin was identified in the mononuclear cells of blood, lymph nodes and spleen several weeks before ulcerative lesions appear. Importantly, diffusion of mycolactone into the blood of *M. ulcerans*–infected mice coincided with alterations in the functions of circulating lymphocytes.

**Conclusion:**

In addition to providing the first evidence that mycolactone diffuses beyond the site of *M. ulcerans* infection, our results support the hypothesis that the toxin exerts immunosuppressive effects at the systemic level. Furthermore, they suggest that assays based on mycolactone detection in circulating blood cells may be considered for diagnostic tests of early disease.

## Author Summary

Mycolactone is a lipophilic molecule produced by *Mycobacterium ulcerans*, the causative agent of the skin disease Buruli ulcer (BU). Mycolactone displays unique cytocidal and immunosuppressive properties that are reflected locally by massive tissue necrosis and minimal inflammation. Here we investigated whether mycolactone diffuses from infected tissues and exerts immunosuppressive properties at the systemic level. We used both a radiolabeled form of the toxin and a direct LC-MS/MS analysis of lipid extracts from internal organs and cell subpopulations to investigate the pharmacodistribution of mycolactone *in vivo*. Using a mouse model of subcutaneous infection with *M. ulcerans*, we show that mycolactone distributes far beyond the sphere of its cytocidal action. The toxin diffused from infected tissues into the blood and the spleen, where it concentrated in mononuclear cell subsets. Importantly, mycolactone was detected in circulating blood several weeks before ulcerative lesions develop. The presence of mycolactone in blood cells was associated with a decreased capacity of circulating lymphocytes to produce interleukin-2 upon stimulation. In addition to providing the first evidence that mycolactone targets key immune cell populations in infected hosts, this work suggests that mycolactone detection in peripheral blood cells may form the basis of diagnostic tests of early disease.

## Introduction

Buruli ulcer (BU) is a cutaneous disease caused by *Mycobacterium ulcerans*, leading to the formation of progressive ulcers, with extensive skin and soft tissue destruction. The presence of a coagulative necrosis forming a nidus for colonies of bacilli, accompanied by minimal inflammation, are considered the most reliable features for the histopathological diagnosis of BU disease [1]. These hallmarks of BU lesions in fact reflect the dual properties of a macrolide toxin produced by *M. ulcerans*, mycolactone, which plays a critical role in bacterial virulence [2],[3]. This unique polyketide has been shown to be a potent cytocidal molecule *in vitro* and *in vivo* [4],[5],[6],[7]. In addition, mycolactone displays significant immunosuppressive properties at non-cytotoxic doses towards a wide range of immune cells [5],[8],[9],[10].

Numerous studies have reported defective IFN-γ responses in BU patients, using assays of PBMC restimulation *ex-vivo* [11],[12],[13],[14],[15],[16]. IFN-γ responses to *M. ulcerans* antigens are reduced in BU patients compared to healthy controls [11],[12],[13],[14], particularly during the early stage of the disease [15],[16]. *M. ulcerans* infection-associated reduction of IFN-γ responses was initially thought to be restricted to mycobacterial antigens. In fact, systemic suppression of IFN-γ responses is not antigen-specific, and resolves after surgical excision of the lesion [17]. Notably, the optimal growth temperature of *M. ulcerans* is below 35°C [18], and animal studies suggest that the bacilli remain essentially localized within ulcerative lesions in subcutaneous tissues [5]. The fact that immunosuppression in BU patients resolves after removal of infected tissues therefore strongly suggests that bacterial factors, such as mycolactone, may diffuse from the bacilli colonies and exert immunosuppressive effects at the systemic level [19].

In the present study, we have investigated the pharmacodistribution of mycolactone following injection in animal models by tracing a radiolabeled form of the toxin *in vivo*, and by directly assessing the integrity and the quantity of mycolactone in lipid extracts from internal organs and cell subpopulations. Our observation that mycolactone diffuses in blood and spleen, and concentrates within distinct immune cellular subsets, supports the notion that mycolactone permits *M. ulcerans* to establish long-term infections by remotely neutralizing the development of cellular immunity.

## Methods

### Animals

Six week old BalB/cByJIco and C57BL/6JIco female mice were purchased from Charles Rivers Laboratories. Mice were housed in a BSL-3 animal facility at the Institut Pasteur, in full compliance with French and European regulation and guidelines on experiments with live animal.

### Mycolactone preparation

*Mu* 1615 wt (ATCC 35840) was obtained from the Trudeau collection. This strain produces a mixture of mycolactones A/B and C [20]. Bacteria were cultivated in Dubos medium complemented with oleic acid-albumin-dextrose 10% (OADC, Becton Dickinson) in spinner flasks at 32°C. To generate ^14^C-labeled mycolactone, exponentially growing cultures were supplemented weekly with 15 µl [1- ^14^C] propionic acid (MP, 40–60 mCi/mmol) and 15 µl [1,2- ^14^C] acetic acid (MP, 50–120 mCi/mmol). After three weeks, mycolactone was purified from bacterial pellets as previously described [2]. The resulting ^14^C-radiolabeled mycolactone showed an activity of 300 cpm/µg. Its biological activity, as measured by the assay described below, was equivalent to that of the unlabeled molecule (data not shown).

### Distribution of radiolabeled mycolactone in blood and organs

BALB/C mice were injected with 300 µg radiolabeled mycolactone via either the sc, ip or iv routes. Animals were bled at the indicated time points, and serum radioactivity monitored by liquid scintillation counting. 24 h post injection, animals were sacrificed and spleen, kidney, liver, brain, fat tissues and intestine were harvested and ground with a Potter-Elvehjem homogeniser in a minimal volume of H_2_O. The radioactivity of the resulting suspensions was measured by liquid scintillation counting.

### Density gradient fractionation

Peripheral blood mononuclear cells (PBMCs) were isolated from whole blood by density gradient centrifugation using Lympholyte-Mammal (Cedarlane, Ontario, Canada) following the instructions of the manufacturer. For PBMC purification from lymph nodes and spleens, organs were first homogenized and passed through nylon filters with 100 µm-diameter pores (Cell Strainer, BD Falcon™), then submitted to density gradient centrifugation using Lympholyte-M (Cedarlane, Ontario, Canada).

### Quantitative analysis of mycolactone presence in mouse organs and cell subsets

Total lipids were extracted from homogenized organs or cell suspensions with chloroform-methanol (2∶1, vol/vol) for 2 h at room temperature. After separation from the aqueous phase, the organic phase was dried and the resulting material resuspended in ice-cold acetone. This acetone-soluble fraction was resuspended in methanol for analysis by ESI-LC-MS and ESI-LC-MS/MS (Helium collision gas), using a Finnigan LCQ ion trap (Thermo Finnigan, USA) coupled with a HP1100 LC fitted with ThermoHypersil BDS C8 column (5 µm, 4.6×250 mm). Mycolactones were eluted with a 40-min gradient from 55 to 95% acetonitrile in water. The presence of *m/z* 765.5 (mycolactone A/B) and *m/z* 749.5 (mycolactone C) was determined by comparison of the MS/MS spectra with those from pure mycolactone preparations. As elution peak areas were directly proportional to mycolactone concentration, 5 µl of a 1 mg/ml reference solution of mycolactone A/B was analyzed with each set of biological samples and used as a standard. The elution peak areas of mycolactones A/B and C were combined to evaluate the total mycolactone concentration in each sample.

### Assay of biological activity

The human T cell line Jurkat subclone E6.1 was cultured in RPMI with 10% FCS, 2 mM L-glutamine, 100 IU/ml penicillin and 100 µg/ml streptomycin. Cells were incubated in microtiter plates (10^5^ cells/well) with serum aliquots (2,5 µl) for 6 h in the presence or absence of 400 ng/ml mycolactone A/B, then activated with 25 ng/ml PMA and 500 ng/ml ionomycin (both from Calbiochem, La Jolla, CA) for 16 h. Culture supernatants were assayed for interleukin (IL)-2 by ELISA (R&D, Minneapolis, MN). For whole blood stimulation assays, 200 µl of pooled blood samples (n = 6) were incubated with anti-CD3 and -CD28 antibodies (both at 10 µg/ml) for 24 h and the production of IL-2 measured by ELISA (R&D, Minneapolis, MN).

## Results

### Pharmacokinetics of mycolactone *in vivo*

**Figure 1:**
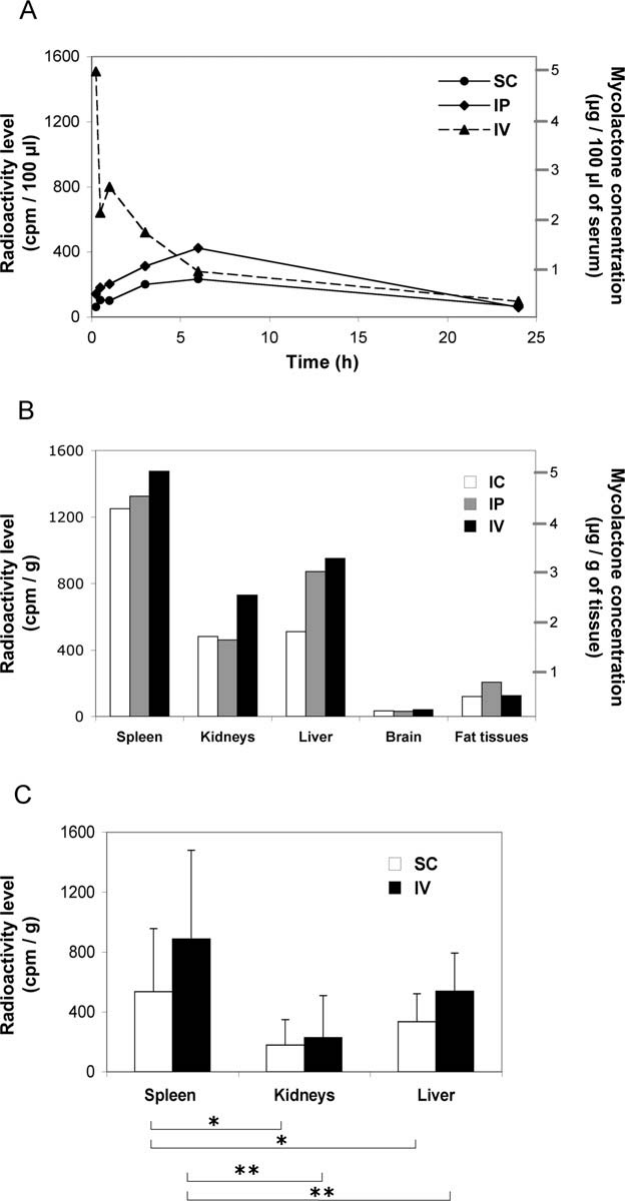
Pharmacokinetics of mycolactone* in vivo.* Panel A shows the kinetics of mycolactone concentration in the circulating blood of mice following an intravenous (IV), intraperitoneal (IP) or subcutaneous (SC) injection of 300 µg of ^14^C-labeled mycolactone. Panel B shows radioactivity levels in various internal organs 24 h post injection of mycolactone via these three administration routes. The left axis indicates cpm levels and the right one the corresponding mycolactone concentrations, for a 300 cpm/µg activity. Data were acquired on pools of blood samples or homogenized organs (n = 3). Panel C illustrates the preferential distribution of mycolactone in the spleen, versus kidney and liver, following injection via the IV (n = 5) or the SC (n = 5) route in three independent experiments. Radioactivity levels in each organ were compared with the Friedman Test (Nonparametric Repeated Measures ANOVA) and Dunn's multiple comparison post-test (*: p<0,05; **: p<0,01).

To investigate the biodistribution of mycolactone, a radiolabeled molecule was generated by feeding *M. ulcerans* cultures with [1- ^14^C] propionic acid and [1,2- ^14^C] acetic acid. Purified ^14^C-labeled mycolactone was injected into mice using three alternative routes of administration, namely subcutaneous (sc), intraperitoneal (ip), or intravenous (iv), and the radioactivity levels in circulating blood were then monitored during 24 hours, after which animals were euthanised (Figure 1A). We found that the blood concentration of iv-delivered mycolactone declined progressively, likely reflecting a distribution outside the vascular system. In contrast, the blood concentration of mycolactone increased slowly following sc or ip delivery. No circulating mycolactone could be detected after 24 hours, for any of the administration routes. The levels of mycolactone in spleen, kidneys, liver, brain and fat tissues were then investigated 24 hours post-injection, by measuring the radioactivity of homogenized organs (Figure 1B). Mycolactone was found in each of the organs except brain, irrespective of the injection route. The relative distribution of mycolactone in spleen, kidney and liver following iv or sc injection was examined. Interestingly, the levels of radioactivity found in the spleen were significantly higher than those of liver and kidney, suggesting that mycolactone displays a relative tropism for this organ (Figure 1C).

### Mycolactone distributes to internal organs without evidence of structural alteration

**Figure 2:**
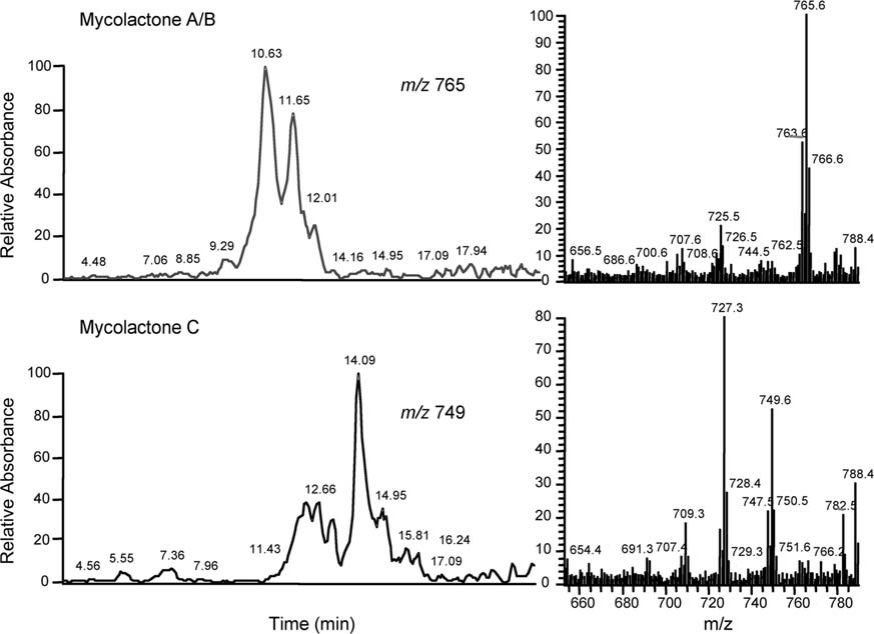
Structural integrity of mycolactone following diffusion into internal organs. The ion trace for *m/z* 765 (Mycolactone A/B) and *m/z* 749 (Mycolactone C), and the corresponding MS/MS spectra are shown for the spleen lipid extract of a mouse injected with mycolactone via the sc route. Data are representative of two independent experiments.

To determine whether mycolactone is present in internal organs as an intact molecule, or as degradation products, we repeated the experiment with equivalent doses of unlabeled mycolactone. Here total lipids were extracted from the various organs and their acetone-soluble fractions were analyzed by LC-MS/MS. In each lipid extract, the typical ion trace of intact mycolactone A/B (*m/z* 765) and C (*m/z* 749) was observed (Figure 2). No evidence that mycolactone had been metabolized by (for example) hydroxylation, methylation, demethylation, double hydroxylation or loss of the side-chain was observed. Furthermore, a quantitative analysis of mycolactone by LC-MS/MS confirmed the relative tropism of mycolactone for the spleen, suggesting that mycolactone may preferentially target this lymphoid compartment (data not shown).

### Mycolactone targets mononuclear cells in blood and spleen cell suspensions

**Figure 3:**
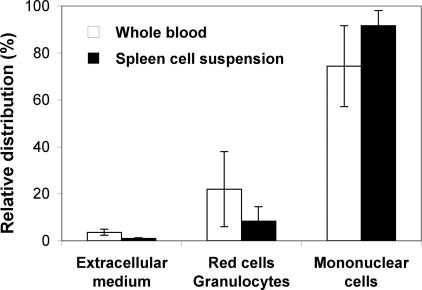
Mycolactone targets mononuclear cells in blood and spleen cell suspensions. The distribution of mycolactone in the gradient density fractions of whole blood or spleen cell suspensions is shown. Mycolactone was added to whole blood (20 µg/ml) or spleen cell suspensions (20 µg/spleen) and incubated for 4 h or 1 h, respectively. The amount of mycolactone distributing in each gradient fractions was then determined by ESI-LC-MS quantitative analysis of their acetone-soluble lipid extracts. Data are mean percentages and SD of duplicate measurements, and are representative of two independent experiments.

The question of mycolactone's tropism was investigated by incubating mycolactone with either blood cells or splenocytes and subjecting the cell suspension to density gradient fractionation. This method allows the purification of mononuclear cells (lymphocytes, monocytes, DCs and macrophages) from extracellular medium, red cells and granulocytes. Strikingly, within 4 hours of incubation with whole heparinized blood, mycolactone (total A/B and C forms) distributed primarily in the mononuclear cell compartment, with only a marginal presence in the extracellular medium and in the red cell/granulocyte fraction (Figure 3). A similar distribution profile was observed when mycolactone was incubated with spleen cell suspensions for 1 hour, strongly suggesting that mycolactone has a particular affinity for mononuclear cell subsets.

### *M. ulcerans*–produced mycolactone diffuses into internal organs of experimentally infected mice

**Figure 4:**
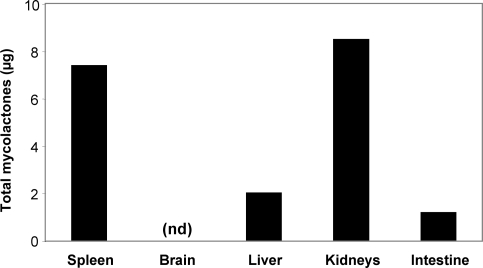
*M. ulcerans*–produced mycolactone diffuses in internal organs of experimentally infected mice. The distribution of mycolactone is shown in internal organs of mice ten weeks post sc infection with 10^4^ *M. ulcerans* bacilli. The amount of mycolactone was determined by ESI-LC-MS analysis of acetone-soluble lipid extracts prepared from pools of 5 homogenized organs. Data correspond to the calculated amount of total mycolactones (A/B and C forms) per organ, nd: not detected.

To evaluate the physiological relevance of this finding, C57BL/6 mice were infected experimentally with *M. ulcerans*, by injection of live bacilli into the tail via the sc route. We have shown previously that this mode of inoculation results in a progressive infection, with multiplication of the bacilli and development of skin ulcerations within 10 weeks following injection of 10^4^ bacilli [21]. Importantly, although bacilli can disseminate to the draining lymph nodes in this model, they do not reach the spleen [5]. As ulcerative lesions developed, mice were sacrificed and the presence of mycolactone was assessed in spleen, brain, liver, kidney and intestine by LC-MS/MS analysis of their acetone-soluble lipid extracts. In accordance with our previous findings with mycolactone-injected mice, mycolactone was detected in spleen, kidneys, and liver but not in the brain (Figure 4). Importantly, mycolactone was structurally intact in these internal organs (data not shown).

### Presence of mycolactone in serum during *M. ulcerans* infection

**Figure 5:**
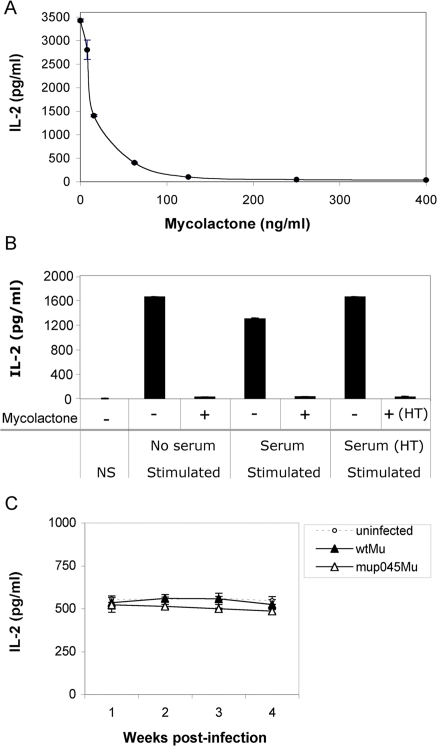
Presence of mycolactone in serum during *M. ulcerans* infection. *A)* The dose-dependent immunosuppressive activity of mycolactone on the IL-2 production of Jurkat T cells is shown. IL-2 concentration was measured in culture supernatants of Jurkat T cells incubated with mycolactone A/B for 6 h prior to stimulation with PMA/ionomycin for 16 h. *B)* The effect of control mouse sera on stimulation-induced production of IL-2 by Jurkat T cells is shown, by comparison to cells incubated in the absence of serum. The graph shows that this inhibitory effect can be neutralized by heating the control mouse sera at 90°C for 10 min (HT), prior to addition onto Jurkat T cells. Controls include cells in the absence of stimulation (NS), and cells activated in the presence of sera spiked with mycolactone (400 ng/ml), then heat-treated. *C)* Immunosuppressive activity of sera harvested from mice infected with wild-type *M. ulcerans* (wtMu) or the mycolactone deficient mutant mup045Mu, as compared to sera from uninfected animals. Data are mean and SD of duplicate measurements of IL-2 production by Jurkat T cells activated in the presence of mouse sera (n = 4), and are representative of two independent experiments.

We investigated the presence of mycolactone in the sera of infected mice indirectly, by taking advantage of its immunosuppressive activity on activation-induced IL-2 production by human lymphocytes [22] (Figure 5A). Here C57BL/6 mice were infected by footpad injection of either the wild-type strain (wtMu), or a mycolactone-deficient strain of *M. ulcerans* (mup045Mu). Sera harvested at different pre-ulcerative stages of the disease were incubated with Jurkat T lymphocytes prior to cell stimulation. Control mouse sera caused a basal inhibition of activation-induced IL-2 production by T cells that was efficiently removed by a heat treatment (Figure 5B). When assessed for their immunosuppressive properties, heat-treated sera from wtMu-infected mice did not differ from those of controls (Figure 5C). Furthermore, no evidence that mycolactone distributes in the sera of wtMu-infected mice could be demonstrated by LC-MS/MS analysis of ethyl acetate extracts, at any stage of the disease (data not shown).

### Mycolactone gains access to mononuclear cell populations during *M. ulcerans* infection

**Figure 6:**
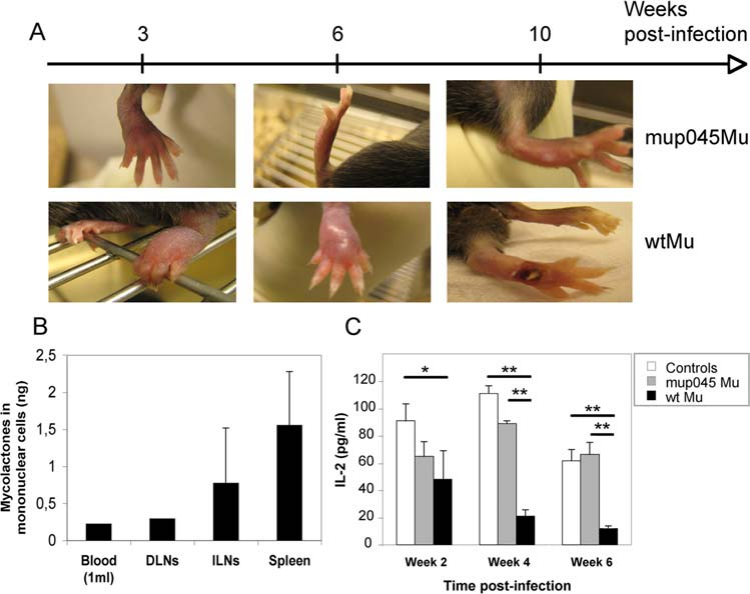
Mycolactone is present in the mononuclear cells of *M. ulcerans*-infected animals. C57BL/6 mice (n = 3) were infected by footpad injection of 10^4^ wtMu or mup045Mu bacilli. The distribution of mycolactone in PBMCs and in the mononuclear cell fraction of draining lymph nodes (DLNs), inguinal lymph nodes (ILNs) and spleens (Spleen) is shown 6 weeks post infection. Mononuclear cells were isolated from pooled samples of whole blood (1 ml), pooled DLNs (n = 3), or from the ILNs and spleens of 3 individual mice. Acetone-soluble lipid were then extracted from cell pellets and mycolactone concentrations determined by quantitative LC/MS-MS analysis. Means and SD are shown for ILNs and spleens. C) IL-2 production after whole blood stimulation with anti-CD3 and -CD28 antibodies for 24 h. Data are mean and SD of IL-2 concentrations, as measured in duplicate for pooled blood samples (n = 4) after 2, 4 and 6 weeks of infection, and are representative of three independent experiments. Differences in IL-2 concentration between groups were analyzed by one-way ANOVA (*: p<0,05; **: p<0,01).

Having shown that mycolactone has a particular affinity for mononuclear cells *in vitro*, we assayed for its presence in this particular cellular subset *in vivo*. Blood samples, lymph nodes and spleens were collected from mice infected with wtMu, or mup045Mu as controls, and cell suspensions submitted to gradient density fractionation. Total lipids were then extracted from mononuclear cell pellets and their acetone-soluble fractions analyzed by LC-MS/MS. In parallel, the functional properties of peripheral blood lymphocytes (PBLs) were evaluated by a whole blood stimulation assay. After 6 weeks of infection with wtMu, that is 4 weeks before ulcerative lesions develop (Figure 6A), mycolactone was detected in PBMCs and in mononuclear cells of the lymph nodes (both draining and distant) and the spleen (Figure 6B). Diffusion of mycolactone into the blood of wtMu-infected mice did not alter the number or the viability of CD4^+^ and CD8^+^ blood T cells (data not shown). However, it correlated with a decreased capacity of PBLs to produce IL-2 in response to stimulation. In contrast, systemic IL-2 responses of mice infected with mup045 Mu were stable and comparable to those of uninfected controls (Figure 6C).

## Discussion

BU is a tropical disease receiving far less attention than TB and leprosy, although it is more common in some endemic regions of West Africa. In contrast to these other two mycobacterioses, BU is acquired from the environment following inoculation of *M. ulcerans* in the dermis by a mechanism involving aquatic niches and insect vectors although the exact mode of transmission remains unknown [3]. BU starts as a painless subcutaneous nodule, oedema or plaque, enlarging over time. As lesions progress, ulcers eventually form that are characterized by an extensive necrosis of subcutaneous tissues accompanied by minimal inflammation [1],[23]. The pathology of the disease is closely associated with the production of a lipophilic toxin, mycolactone. This macrocyclic polyketide is highly cytotoxic to a variety of mammalian cells *in vitro*, and the injection of mycolactone into the skin of guinea pigs is sufficient to provoke ulcers [2]. Although the presence of mycolactone is reflected locally by its damaging effects on infected tissues any investigation of its diffusion outside the ulcerative lesion has been rendered difficult by the lack of a detection tool for this poorly immunogenic compound. In the present study, we have used a radiolabeled mycolactone to show that the toxin diffuses far beyond the sphere of its cytocidal action at the site of inoculation, or at the point of *M. ulcerans* infection.

Mycolactone is cytotoxic and pro-apoptotic at micromolar concentrations, but in addition non-cytotoxic doses in the nanomolar range can efficiently suppress the functional biology of several types of mononuclear cells. Mycolactone was shown to inhibit the activation-induced production of IL-2 by human lymphocytes, and of TNF by monocytes and macrophages [5],[9]. Mycolactone also blocked the capacity of dendritic cells (DCs) to prime cellular responses and to produce chemotactic signals of inflammation [8]. Lymphocytes, monocytes, DCs and macrophages compose the mononuclear cell fraction of blood and lymphoid organs. Together, these cell populations contribute to the generation of innate and acquired cellular immune responses, which are critical for protective immunity against mycobacterial infections. The fact that mycolactone targets mononuclear cells in mice infected with *M. ulcerans* strongly suggests that these cell subsets are immunosuppressed by the toxin *in vivo*.

The organ distribution of mycolactone revealed a relative tropism of this molecule for the spleen, which is similar to that of other lipophilic immunosuppressive compounds such as rapamycin or FK506 [24],[25]. However these two drugs are metabolized in the liver, which produces a large array of metabolites [26],[27]. Surprisingly, mycolactone was preserved in all the organs (including the liver) that we analyzed by LC-MS/MS, and no metabolite could be identified. Further studies, for example the evaluation of toxin levels in bile, faeces and urine, will be required to determine how infected animals eliminate mycolactone.

In contrast to FK506 and rapamycin, which concentrate primarily in erythrocytes (>90%) and only minimally in lymphocytes (<1%) in circulating blood, we found that mycolactone concentrates in mononuclear cells [24],[28]. This is true both for whole blood and for splenocyte cell suspensions, which are richer in lymphocytes. FK506 and rapamycin are structural analogues binding the same intracellular receptor FKBP12, although the resulting complex targets a different molecule. Their sequestration by red blood cells is explained by the high immunophilin levels of erythrocytes. For mycolactone the molecular target and pathway of action of the toxin are still unknown. Our findings, combined with the observation that mycolactone is a potent immunosuppressor of monocytes, macrophages, DCs and lymphocytes suggest that the molecular target of mycolactone may be expressed preferentially by mononuclear cells.

We detected structurally intact mycolactone in PBMCs 6 weeks post infection with *M. ulcerans*, that is 4 weeks before ulcerative lesions start to develop in this mouse model. This is the first evidence that mycolactone diffuses outside the lesions of an organism infected with *M. ulcerans* and circulates via peripheral blood. BU is often diagnosed on the basis of clinical findings, because laboratory diagnoses based on smear examination, *M. ulcerans* cultures, or PCR detection require significant logistics and equipment. Simple and rapid diagnostic field tests for BU are urgently needed for this disease to be treated locally and inexpensively. Our results obtained in the mouse model suggest that assays based on mycolactone detection in circulating blood cells may be considered for diagnostic tests of early disease. We are currently trying to define ways to detect mycolactone directly in the blood cells of BU patients, which may be applicable to diagnosis in field conditions.
